# Dyslipidaemia was correlated to the posterior circulation infarction in non-diabetic populations

**DOI:** 10.1186/s12944-018-0799-0

**Published:** 2018-06-26

**Authors:** Yun Luo, Zheng Li, Jiahui Zhang, Jingwei Li, Zhengjuan Lu

**Affiliations:** 1 Department of Neurology, Affiliated Drum Tower Hospital of Nanjing University Medical School, No. 321 Zhongshan Road, Gulou District, Nanjing, 210008 Jiangsu Province China; 2 Department of Rehabilitation Medicine, Affiliated Drum Tower Hospital of Nanjing University Medical School, No. 321 Zhongshan Road, Gulou District, Nanjing, 210008 Jiangsu Province China

**Keywords:** Triglyceride, High density lipoprotein cholesterol, Acute ischemic stroke, Posterior circulation infarction, Diabetes mellitus

## Abstract

**Background:**

Diabetes mellitus (DM) was prone to happening in posterior circulation infarction (POCI) and DM also has the impact on the lipids, our study was to investigate the correlation between lipid compositions and POCI.

**Methods:**

Data was collected from the patients with acute ischemic stroke (AIS) hospitalization in Affiliated Drum Tower Hospital of Nanjing University Medical School from October 2008 to May 2012. Lipids and other risk factors in the different populations were investigated in relation to occurrence of POCI based on the infarction location.

**Results:**

Six hundred ten patients with AIS were included in this study, which had 428 with anterior circulation infarction (ACI) and 182 with POCI. Elevated Triglyceride (TG) and decreased High density lipoprotein cholesterol (HDL-C) were seen in the POCI of total populations and AIS without DM compared to the ACI, but not in the populations of AIS with DM, so did the elevated TG/HDL-C ratios. Also, the percent of low HDL-C level and high TG level were higher in POCI group than that in ACI group. Furthermore, single factors logistic regression demonstrated that TG, HDL-C and TG/HDL-C ratio were correlated to the POCI whatever in the total populations or AIS without DM, but this kind of trend just maintained in the populations of AIS without DM after adjusting by relative interference factors.

**Conclusion:**

Dyslipidaemia was prone to happening in POCI compared to ACI in the non-diabetic populations, which was correlated to the pathogenesis of POCI.

## Background

Acute ischemic stroke (AIS) was divided into anterior circulation infarction (ACI) and posterior circulation infarction (POCI) according to the infarction location, a difference was made between ACI and POCI in terms of clinical manifestations and prognosis. Despite POCI just accounting for 20% of AIS, and it would have a relatively good outcome, but it had high rate of recurrence. About the risk factors, was there has any difference between ACI and POCI, the conclusions was inconsistent.

As the important risk factors of AIS, diabetes mellitus (DM) had obvious impact on both of subtypes, but DM was more prone to linked with POCI [1], which was identified in many studies include ours (Article in Chinese) [2–4]. A study from China demonstrated, not only diabetes mellitus, but also male gender was associated with greater likelihood of POCI than ACI [5].

Diabetes mellitus had obvious impact on the lipids, which mainly reflects in the rise of Triglyceride (TG) and fall of High density lipoprotein cholesterol (HDL-C) [6, 7]. Both the blood glucose and lipids would play the important role on the occurrence of arteriosclerosis, while lipids would play the more direct role [8, 9]. Did this mean that the induction of arteriosclerosis and ischemic stroke by DM was through lipids, and this was proved in our previous study, that was, low level of HDL-C was correlated to the occurrence of AIS induced by DM [10], which demonstrated that dyslipidaemia was prone to happening in the AIS with DM.

There was more percentage of DM in the POCI, also, DM would have the impact on the expression of lipids. Based on the background above, we wonder if there would have more dyslipidaemia in the POCI compared to the ACI, which maybe act as the important pathogenic factors of POCI.

## Methods

### Study subjects

Data for this retrospective study was collected from the hospitalization patients of department of neurology in Affiliated Drum Tower Hospital of Nanjing University Medical School from October 2008 to May 2012, AIS was defined as symptom onset within7 days. The study was approved by our institutional committee. Patients who were found with pre-stroke impairment or insulin-dependent diabetes mellitus were excluded. At admission, plain CT scan of the head was done to rule out haemorrhage and MRI was done to identify the new infarction and the location of the lesion, otherwise such patients would also be excluded. The stroke subtype (ACI and POCI) was defined based on the classification of Bogousslavsky’s [11].

### Definition of vascular risk factors

Hypertension and DM were defined as participants with history of relative disease or new diagnosis according to the China hypertension and DM standard (just non-insulin-dependent diabetes were included), while atrial fibrillation (AF) was defined as participants with history of AF or new diagnosis by electrocardiogram.

### Blood collection and analysis

Venous blood was collected following overnight fasting for at least 12 h, and analyzed by a solid-phase chemiluminescent immunometric assay on Immulite 2000 with the manufacturer’s reagents as directed to detect total bilirubin (Tbil), direct bilirubin (Dbil), blood glucose (BG), uric acid (UA), TG, total cholesterol (TC), HDL-C, Low density lipoprotein cholesterol (LDL-C).

### Statistical analyses

Statistical analyses were performed with SPSS 17.0 software. The results are expressed as constituent ratio for categorical variables (χ2 test) and as mean ± SEM for the continuous variables (t-test) depending on their normal distribution. The level-risk relationship was expressed as an OR, with a corresponding 95% CI, through logistic regression. Level of significance for statistical purposes was stated at *p* < 0.05.

## Results

### Baseline characteristics

Six hundred ten patients with AIS were included in the trial, among them, 385 were male and 225 were female, whose age range from 15 to 92. There were 202 patients who had DM coexisted with AIS. Infarction happened to the ACI was 428, and POCI was 182.

### Risk factors of POCI compared to the ACI

There was 29% of DM in the ACI, while 42.9% in the POCI, the difference was significant (*P* = 0.001), the same trend was existed in hypertension and BG. While AF and Dbil were more prevalent in ACI. Also, the difference of TG and HDL-C between ACI and POCI was significant, as shown in Table 1.

**Table 1:** Comparison of risk factors between ACI and POCI

Variable	ACI	POCI	*P*
Male (%)	61.4	67.0	0.200
Age	67.131 ± 0.633	65.758 ± 0.935	0.232
DM (%)	29.0	42.9	0.001
Hypertension (%)	67.8	79.1	0.005
AF (%)	15.7	7.7	0.009
Dbil	4.867 ± 0.130	4.329 ± 0.156	0.017
Tbil	18.638 ± 0.462	17.548 ± 0.674	0.192
BG	6.758 ± 0.131	7.297 ± 0.227	0.041
UA	326.348 ± 4.751	335.524 ± 8.334	0.313
CRP	5.448 ± 0.873	3.272 ± 0.831	0.072
TG	1.450 ± 0.922	1.641 ± 1.116	0.028
TC	4.795 ± 0.981	4.809 ± 1.142	0.877
HDL-C	1.170 ± 0.367	1.082 ± 0.346	0.006
LDL-C	2.549 ± 0.733	2.595 ± 0.779	0.485

### Comparison of lipids level between ACI and POCI in the populations of AIS with and without DM

DM would have the impact on express of lipids [12], which had been proved in a series of studies include ours [13]. Due to more DM coexisted with POCI, we wonder if this kind of distribution difference resulted in difference of the lipids between ACI and POCI. But to our surprised, elevated TG and decreased HDL-C were just seen in the populations of AIS without DM, while not in the AIS with DM (Table 2).

**Table 2:** Comparison of lipid composition between ACI and POCI

Variables	ACI(*n* = 124)	POCI(*n* = 78)	*P* value
With DM			
TG(mmol/l)	1.555 ± 1.021	1.623 ± 0.799	0.617
TC(mmol/l)	4.722 ± 1.118	4.858 ± 1.169	0.409
HDL-C(mmol/l)	1.076 ± 0.327	1.053 ± 0.321	0.629
LDL-C(mmol/l)	2.530 ± 0.832	2.687 ± 0.810	0.190
Without DM			
TG(mmol/l)	1.407 ± 0.877	1.655 ± 1.308	0.030
TC(mmol/l)	4.825 ± 0.919	4.773 ± 1.126	0.639
HDL-C(mmol/l)	1.209 ± 0.375	1.103 ± 0.363	0.013
LDL-C(mmol/l)	2.557 ± 0.689	2.526 ± 0.752	0.707

### Comparison of lipids distribution between the ACI and POCI

**Fig. 1:**
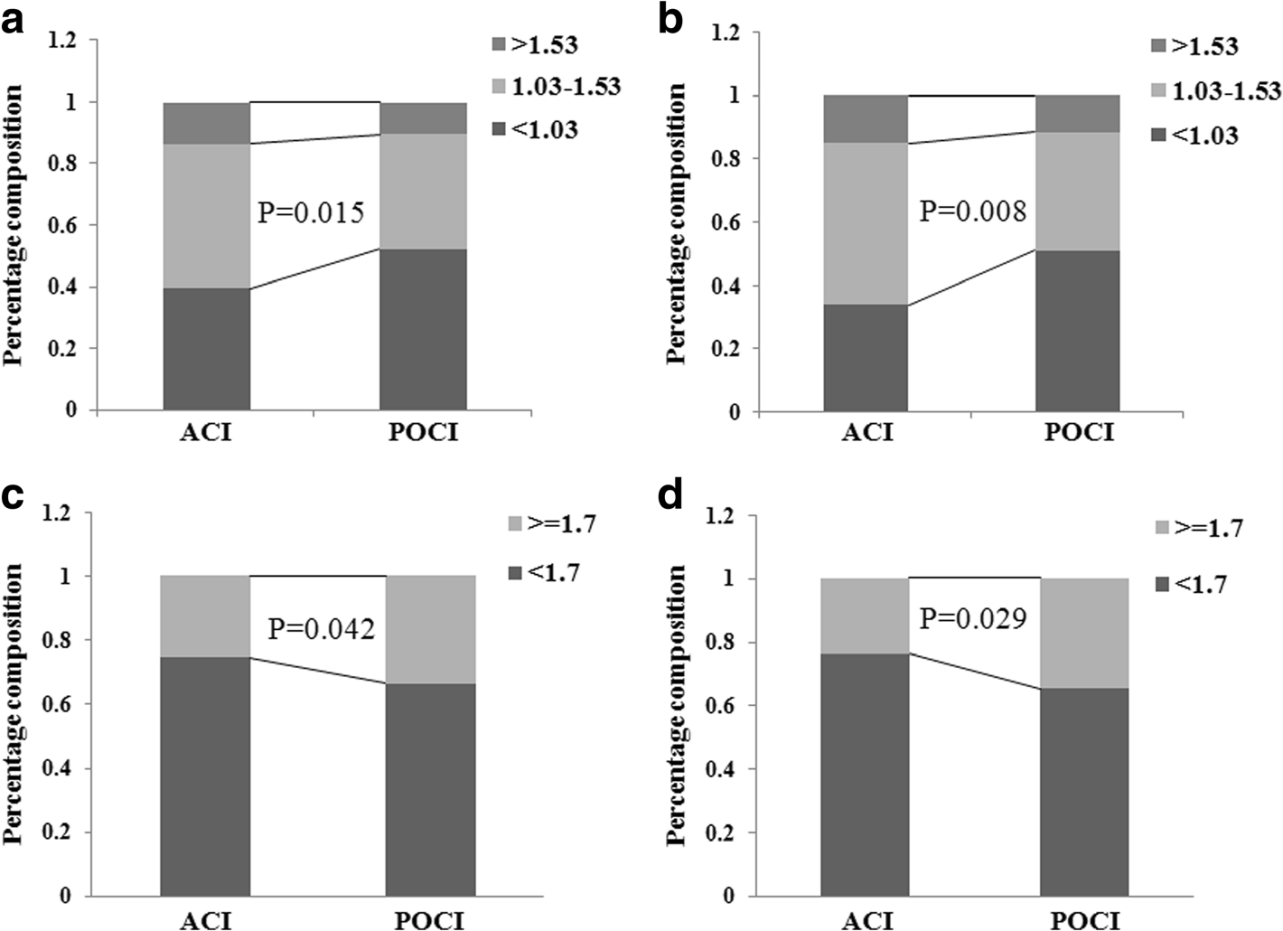
Distribution of HDL-C and TG levels in the total and non-DM populations. **a** HDL-C in the total populations, **b** HDL-C in the non-DM populations, **c** TG in the total populations, **d** TG in the non-DM populations)

HDL cholesterol concentrations were grouped into 3 levels: < 1.03, 1.03–1.53 and > 1.53 mmol/l. We analyzed the difference of distribution of HDL-C between the POCI and ACI whenever in the total or AIS without DM populations, and found that, the percent of low HDL-C level was higher in POCI group than that in ACI group. Also, TG concentrations were grouped into 2 levels: < 1.7 and > 1.7 mmol/l, we found, the percent of high TG level was higher in POCI group than that in ACI group both in the total or AIS without DM populations (Fig. 1).

### Difference of the ratio of TG/HDL-C between ACI and POCI

**Fig. 2:**
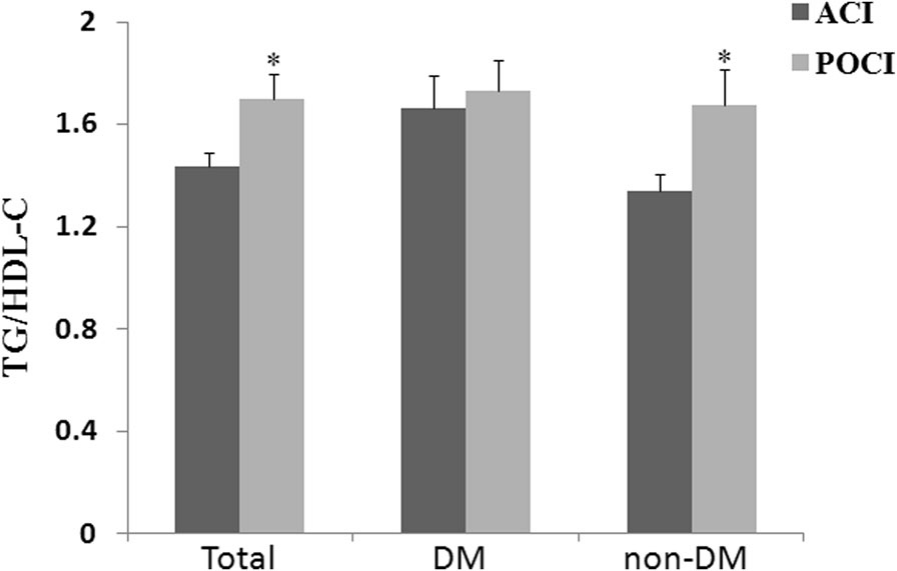
Comparison of TG/HDL-C ratios between ACI and POCI in different populations (^*^P < 0.05 vs *ACI*)

Dyslipidaemia in the POCI was mainly resulted from the change of TG and HDL-C, while the ratio of TG/HDL-C could be used as the marker in prognosis of vascular events [14], so we compared the ratio of TG/HDL-C between ACI and POCI. We found that TG/HDL-C ratios was higher in the POCI compared to the ACI both in the total and non-diabetic populations, which demonstrated that dyslipidaemia played the more important role on POCI (Fig. 2).

### Dyslipidaemia was not correlated to POCI in the total AIS populations

All the patients of AIS, were divided into two groups named ACI and POCI based on the location of infarction. To study the risk factors of POCI, we first performed the single-factor logistic regression, and found that TG, HDL-C and TG/HDL-C ratio were correlated to the POCI, but after multivariable logistic regression analysis, the correlation was disappeared (Table 3).

**Table 3:** Relative risk of POCI versus risk factors in the total populations of AIS

Variables	Beta estimate	OR	95% CI	*P* value
Model 1: unadjusted				
Male	0.243	1.276	0.885–1.838	0.191
Age	−0.008	0.992	0.979–1.005	0.231
DM	0.609	1.839	1.282–2.636	0.001
Hypertension	0.590	1.803	1.196–2.720	0.005
AF	−0.801	0.449	0.245–0.822	0.009
Dbil	−0.104	0.902	0.828–0.981	0.017
Tbil	−0.013	0.987	0.968–1.007	0.193
UA	0.001	1.001	0.999–1.003	0.313
TG	0.186	1.204	1.015–1.428	0.033
TC	0.013	1.013	0.856–1.199	0.876
HDL-C	−0.743	0.476	0.280–0.808	0.006
LDL-C	0.083	1.086	0.862–1.369	0.484
TG/HDL-C	0.169	1.184	1.033–1.357	0.015
Model 2: adjusted for Hypertension, DM, AF, Dbil				
TG	0.108	1.114	0.934–1.328	0.229
HDL	−0.522	0.594	0.343–1.028	0.063
TG/HDL-C	0.097	1.102	0.956–1.271	0.180

### Dyslipidaemia was correlated to the POCI in the AIS without DM populations

Populations of AIS without DM were also divided into ACI and POCI, logistic regression demonstrated that TG, HDL-C and TG/HDL-C ratio were correlated to the POCI, whatever in the single-factor or multivariable logistic regression (Table 4).

**Table 4:** Relative risk of POCI versus risk factors in the populations of non-diabetic AIS

Variable	Beta estimate	OR	95% CI	*P* value
Model 1: unadjusted				
Male	0.453	1.574	0.967–2.562	0.068
Age	−0.009	0.991	0.976–1.008	0.296
Hypertension	0.626	1.870	1.127–3.102	0.015
AF	−0.436	0.647	0.322–1.300	0.221
Dbil	−0.105	0.901	0.808–1.004	0.058
Tbil	−0.017	0.983	0.958–1.008	0.180
BG	−0.022	0.978	0.846–1.131	0.765
UA	0.001	1.001	0.999–1.003	0.222
TG	0.222	1.248	1.011–1.541	0.039
TC	−0.055	0.946	0.752–1.191	0.638
HDL-C	−0.868	0.420	0.211–0.834	0.013
LDL-C	−0.061	0.941	0.685–1.292	0.707
TG/HDL-C	0.220	1.247	1.044–1.488	0.015
Model 2: adjusted for Hypertension				
TG	0.592	1.807	1.086–3.006	0.023
HDL	−0.809	0.445	0.221–0.895	0.023
TG/HDL-C	0.203	1.225	1.024–1.466	0.027

## Discussion

Our investigation was a retrospective study of patients with AIS in which the correlation between POCI and lipids was evaluated. We first confirmed that there was more percentage of DM in the POCI compared to the ACI, while DM would have the impact on the expression of lipids, which has been identified in our previous study [13]. We hypothesize that this kind of distribution difference would result in difference of lipids between ACI and POCI, but this hypothesis was uncorrected. In fact, the significant difference of TG, HDL, TG/HDL-C between ACI and POCI were just existed in the populations of non-diabetic AIS, which was correlated to the pathogenesis of POCI.

The association between DM and POCI has been proved in previous studies [15, 16]. In a prospective study in Koreans, DM was an independent risk factor for intracranial atherosclerosis only in posterior circulation [2]. These researchers speculated that the effect of metabolic disorders was more related to the POCI, which indicated the differing neurovascular origins of POCI and ACI [3]. In our research, we found that more DM coexisted with POCI compared to the ACI, which seemed to demonstrate that DM cause dyslipidaemia and finally increase the incidence of POCI.

Dyslipidaemia was prone to happening in the diabetic populations [17, 18], which played important role in the pathogenesis of AIS. Insulin resistance (IR) existed in many diabetic population and altered the lipids and lipoprotein metabolism [6, 19, 20]. Through a series of signaling pathways adjusting, the decrease in HDL available for participation in reverse cholesterol transport may finally caused the atherogenicity in AIS [21]. In the prevention of AIS linked to the DM, management of lipids was important as well as the glucose control [22]. Compared to the diabetic populations, what we should do in the non-diabetic populations? There did not involve the glucose control, but dyslipidaemia also act as an important risk factors, mainly in the POCI, which was identified in this study.

Low levels of HDL-C had been proved correlated with AIS [23–25], but the difference between POCI and ACI was unclear. In our study, elevated TG/HDL-C ratio was prone to happening in the POCI compared to the ACI, both in the total and non-diabetic populations, while not in the populations of DM. Why was this kind of manifest? We found, level of TG was higher and HDL-C was lower in the POCI of total and non-diabetic patients, resulting in higher TG/HDL-C ratios compared to the ACI, while the level of TG and HDL-C had no difference between POCI and ACI in the diabetic patients.

Perhaps this kind of infarction location difference also shown difference of the expression of HDL-C and TG, but compared to the influence of DM, the former is negligible, and finally determined that there had no difference of HDL-C and TG between ACI and POCI in the diabetic patients. In order to confirm whether our hypothesis was right, we studied the risk factors of POCI in total and patients without DM.

We observed the elevated TG/HDL-C ratio in the POCI rooted from the rise of TG and fall of HDL-C, then, logistic regression demonstrated it was correlated to the POCI just in the non-diabetic populations, which indicated that elevated TG/ HDL-C ratio was an important risk factor in the pathogenesis of POCI in the non-diabetic populations. Elevated TG and decreased HDL-C were thought to be asatherogenic dyslipidaemia [26] and the key metabolic abnormalities in insulin resistance (IR) states [27], which was familiar in the patients of DM. This result we found perhaps predicate that more patients of POCI had been in the condition of pre-diabetes.

## Conclusion

Our current study provided the evidence that high level of TG and low level of HDL-C, furthermore, elevated TG/HDL-C ratio, were correlated to the occurrence of POCI in the non-diabetic populations. We would certainly give enough attentions to the dyslipidaemia in the diabetic patients [28], but to prevent the occurrence of ischemic stroke in the non-diabetic populations, more attentions should be paid to the change of the lipids including TG, HDL-C and TG/HDL-C ratio, which has the obvious correlation with POCI.

## Abbreviations

ACI: Anterior circulation infarction
AF: Atrial fibrillation
AIS: Acute ischemic stroke
BG: Blood glucose
Dbil: Direct bilirubin
DM: Diabetes mellitus
HDL-C: High density lipoprotein cholesterol
LDL-C: Low density lipoprotein cholesterol
POCI: Posterior circulation infarction
Tbil: Total bilirubin
TC: Total cholesterol
TG: Triglyceride
UA: Uric acid
